# Global labor flow network reveals the hierarchical organization and dynamics of geo-industrial clusters

**DOI:** 10.1038/s41467-019-11380-w

**Published:** 2019-08-01

**Authors:** Jaehyuk Park, Ian B. Wood, Elise Jing, Azadeh Nematzadeh, Souvik Ghosh, Michael D. Conover, Yong-Yeol Ahn

**Affiliations:** 1 School of Informatics, Computing, and Engineering, Indiana University, Bloomington, IN 47408 USA; 2 LinkedIn, Sunnyvale, CA 94043 USA; 3 S&P Global, New York, NY 10004 USA; 4 Workday, Inc, Pleasanton, CA 94588 USA

**Keywords:** Information theory and computation, Business, Information technology, Information technology, Information theory and computation

## Abstract

Groups of firms often achieve a competitive advantage through the formation of geo-industrial clusters. Although many exemplary clusters are the subjects of case studies, systematic approaches to identify and analyze the hierarchical structure of geo-industrial clusters at the global scale are scarce. In this work, we use LinkedIn’s employment history data from more than 500 million users over 25 years to construct a labor flow network of over 4 million firms across the world, from which we reveal hierarchical structure by applying network community detection. We show that the resulting geo-industrial clusters exhibit a stronger association between the influx of educated workers and financial performance, compared to traditional aggregation units. Furthermore, our analysis of the skills of educated workers reveals richer insights into the relationship between the labor flow of educated workers and productivity growth. We argue that geo-industrial clusters defined by labor flow provide useful insights into the growth of the economy.

## Abstract (web summary)

There is a lack of systematic approaches to identify and analyze the hierarchical structure of geo-industrial clusters at the global scale. Here the authors use LinkedIn's employment history data to construct a global labor flow network from which they find that the resulting geo-industrial clusters exhibit a stronger association between the influx of educated-workers and financial performance compared to existing aggregation units.

## Introduction

Why are the leading internet companies located near each other in Silicon Valley? Why do aspiring actors who dream of stardom move to Hollywood? Even though modern telecommunication technologies allow remote collaboration and many companies are no longer restrained by physical supply chains, numerous and conspicuous geo-industrial clusters concentrate within small geographical areas. Such geographical agglomeration of interconnected firms, or Clusters^1,2^, is a key conceptual framework for policymakers and business economists, from global organizations such as the OECD^3^ and the World Bank^4,5,^ to regional development agencies in national governments^6^.

However, existing studies on geo-industrial clusters are challenged with the following limitations. First, the concept of the geo-industrial cluster is vague, and the considered range of spatial and industrial proximity greatly varies across studies^6^. The lack of a concrete definition hampers the systematic analysis of empirical data, as well as the creation of a solid policy model. This is exacerbated by the lack of extensive empirical data, limiting most studies to focus on a small number of exemplars and encouraging reliance on the top–down approach^7–10^, where scholars or policymakers subjectively, although with expertise, assign an industrial sector code to a set of selected administrative districts^11^. As clusters primarily arise from the strategic decisions of firms^1,2^, such top–down approach based on predefined industrial and regional codes may fail to capture the organic and emergent nature of clusters and their dynamics. Finally, the isolated case-based studies constrain researchers from investigating the connections between clusters as well^1,2,7,9^.

**Fig. 1:**
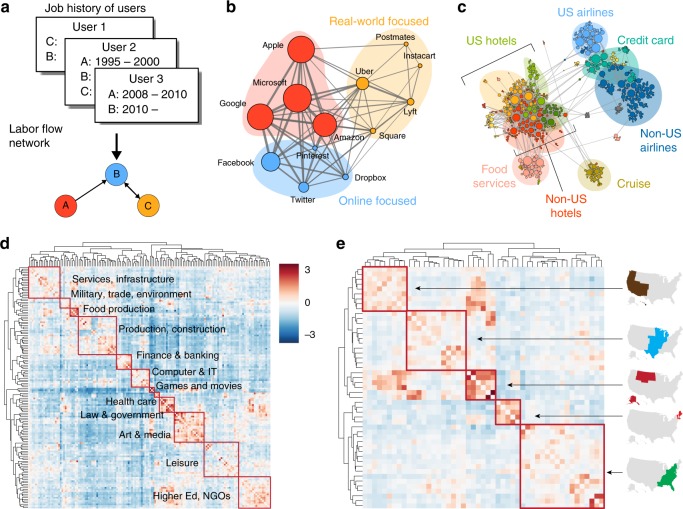
The structure of the labor flow network is shaped by firms’ geographic proximity and talent demands. **a** A labor flow network is comprised of organizations (nodes) and the flows of people between them (directed, weighted edges) as defined by historical records of job changes. **b**, **c** Two illustrative examples of geo-industrial clusters defined as hierarchically-organized geo-industrial clusters in the labor flow network with high intra-cluster talent mobility. **b** Within a cluster of software & internet technology firms, we see sub-clusters with respect to types of services—online (blue), offline (yellow), and both (red), which are also linked to the age of firms. **c** Geo-industrial clusters shaped by geography and area of specialization are also evident within a cluster of travel-related firms. **d**, **e** A transition matrix of labor flows between LinkedIn users’ self-reported industries (normalized with the expected transition volume) highlights labor flows within and between macroeconomic sectors. The effect of geographic proximity on labor mobility is also evident in the matrix of labor flows between US states (see Methods for details)

Here, by proposing an organic way to identify geo-industrial clusters from a labor flow network, we reveal the hierarchical organization of geo-industrial clusters across multiple scales in the global economy and argue that examining their interconnected hierarchical structure is a critical step towards understanding their role in broader economic contexts. Our approach to identify geo-industrial clusters and their hierarchical organization involves identifying concentrated labor flow between firms (see Fig. 1a). The job transitions of workers, labor flow, is central in driving firms to form geo-industrial clusters thanks to knowledge spillover and labor market pooling^12–14^. Labor flow thus provides crucial clues to the identification of geo-industrial clusters^15,16^. To map these geo-industrial clusters we leverage LinkedIn’s data set, which documents the professional demographics and employment histories of >500 million individuals between 1990 and 2015. This data set allows us to create, to our knowledge, the largest global labor flow network^15–19^ yet analyzed. The network consists of directed, weighted edges capturing ~130 million job transitions between more than four million firms. We show that the structure of this global labor flow network reveals the multi-scale hierarchical organization of geo-industrial clusters, which constitute a natural, emergent unit of analysis for the global economy.

## Results

### General patterns in labor flow

Workers tend to change their jobs between geographically close firms with similar skill requirements^20–23^. This tendency leads to knowledge spillover and innovation, serving as a prominent feedback mechanism in the formation of geo-industrial clusters^9,24–27^. As geo-industrial clusters form, they also affect labor flow by attracting the workers with pertinent skills, creating a strong concentration of skills and knowledge locally. This feedback, where geo-industrial clusters and workers are influencing each other, produces concentrated job movements, which in turn can be leveraged to identify clusters as network communities, groups of cohesively interconnected nodes on a network^28,29^; in a labor flow network, the cluster of firms would manifest as network communities, tied together by concentrated labor flow (see Fig. 1).

From our data, relevant geo-industrial clusters can easily be found across domains, from technology firms of distinct flavors and ages (Fig. 1b) to clusters of travel and hospitality industries (Fig. 1c), which are concentrated with respect to both specialization (e.g., airlines, promotional credit cards, food service, or cruise lines) and geography. The hierarchical structure of these geo-industrial clusters is evident in the makeup of the non-US airline geo-industrial cluster, which, itself, is comprised of smaller sub-modules corresponding to serving geographically distinct markets such as Europe and the Middle East.

The concentration based on the industrial and geographic proximity can be separately observed through an industry-wise and a region-wise transition matrix. We calculate two normalized transition matrices between industries and US states respectively (Fig. 1d, e; see Methods and Supplementary Note 1 for details). Industries are split into two large clusters, which roughly correspond to production (upper left) and public and consumer services (bottom right). In the context of the three-sector theory^30,31^, or rather a more recent four-sector framework^32^, the upper-left cluster is organized around the primary, secondary, and some of the tertiary sector (infrastructure and business support), whereas the bottom-right cluster consists of industries mostly in the quaternary sector, including higher education, government, law, healthcare, leisure, and media (see Supplementary Figure 1 for all original industry labels). Although finance and information technology are often classified into the quaternary sector, here they are clustered with production and manufacturing, highlighting their strong connection to engineering and production. Retail, on the other hand, is clustered more closely with other quaternary services, as opposed to tertiary services.

The abundance of off-diagonal interactions emphasizes the complex interconnected nature of the economy. For instance, the law and government sectors are more likely to generate a cluster with military, trade, and environment sectors than other sectors of the economy, although such connections cross the boundary of the two largest industry clusters. Curiously, the leisure industry is one of the most widely connected, exhibiting strong connections to many other sectors, including healthcare, education, art, media, and manufacturing. The labor flow network also displays strong geographical clustering, as shown in Fig. 1e.

### Industry versus geography

The clear presence of clustering with respect to both industry and geography prompts the following questions: which factor is more important in determining the structure of geo-industrial clusters, industry, or geography? How do these factors shape the hierarchical structure of these clusters? If the composition of a geo-industrial cluster is heavily constrained by industrial or geographical proximity, we expect to see clusters form around an industry or a location, respectively. Therefore, measuring cluster homogeneity in terms of industry and region not only allows us to evaluate the validity of clustering but also allows us to estimate the strength of each constraint. In doing so, we assess the relevance of the clusters as well as the strength of industrial or geographical constraints.

**Fig. 2:**
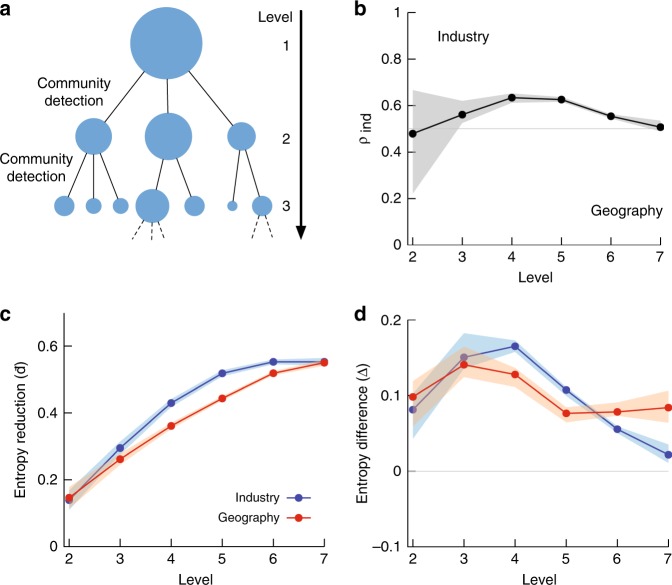
The impact of geography and industry across scales. The top-level communities of this network found through the Louvain method (see Methods) have a modularity of 0.47. **a** We recursively apply network community detection to discover the labor flow network’s hierarchical structure. See Methods for more details. **b** Both industry and geography affect job transition across all scales, but industry has a more important role in the middle of the hierarchy as seen by the proportion of communities with a greater reduction in industry entropy (*ρ*_ind_). **c** The average reduction of metadata entropy $\ {\bar{{\!\!}\boldsymbol d}}$ (see Methods for the definition) at each level of the hierarchical community structure, calculated with respect to the whole network. The monotonic increase indicates that smaller communities are more homogeneous as expected. **d** This entropy reduction is greater than expected by a null model. The difference between the observed entropy reduction and the reduction in a randomized hierarchical null model is denoted as Δ. Positive Δ indicates that the homogeneity of clusters is stronger than expected

We quantify the homogeneity of network communities by calculating the Shannon entropy of cluster feature vectors that document the fraction of people in the geo-industrial cluster who belong to each industry or region (see Methods). We quantify the relative importance of industry and geography by calculating the ratio between the number of geo-industrial clusters at each level with a greater reduction in industrial entropy and those with a greater reduction in geographical entropy. Our measurement in Fig. 2b, c shows that the industry tends to play a more important role than geography in constraining labor flow and its strength is strongest at the middle of the hierarchy (see Methods, Supplementary Figure 2, and Supplementary Note 2 for more details). In other words, network communities tend to be broken down into smaller communities mainly based on industrial categories. As shown in Fig. 2d, the average entropy reduction is larger than expected by chance throughout the hierarchy, indicating that the identified clusters are cohesive and meaningful. Then, how are they organized within the global network?

### Hierarchical structure of the labor flow network

**Fig. 3:**
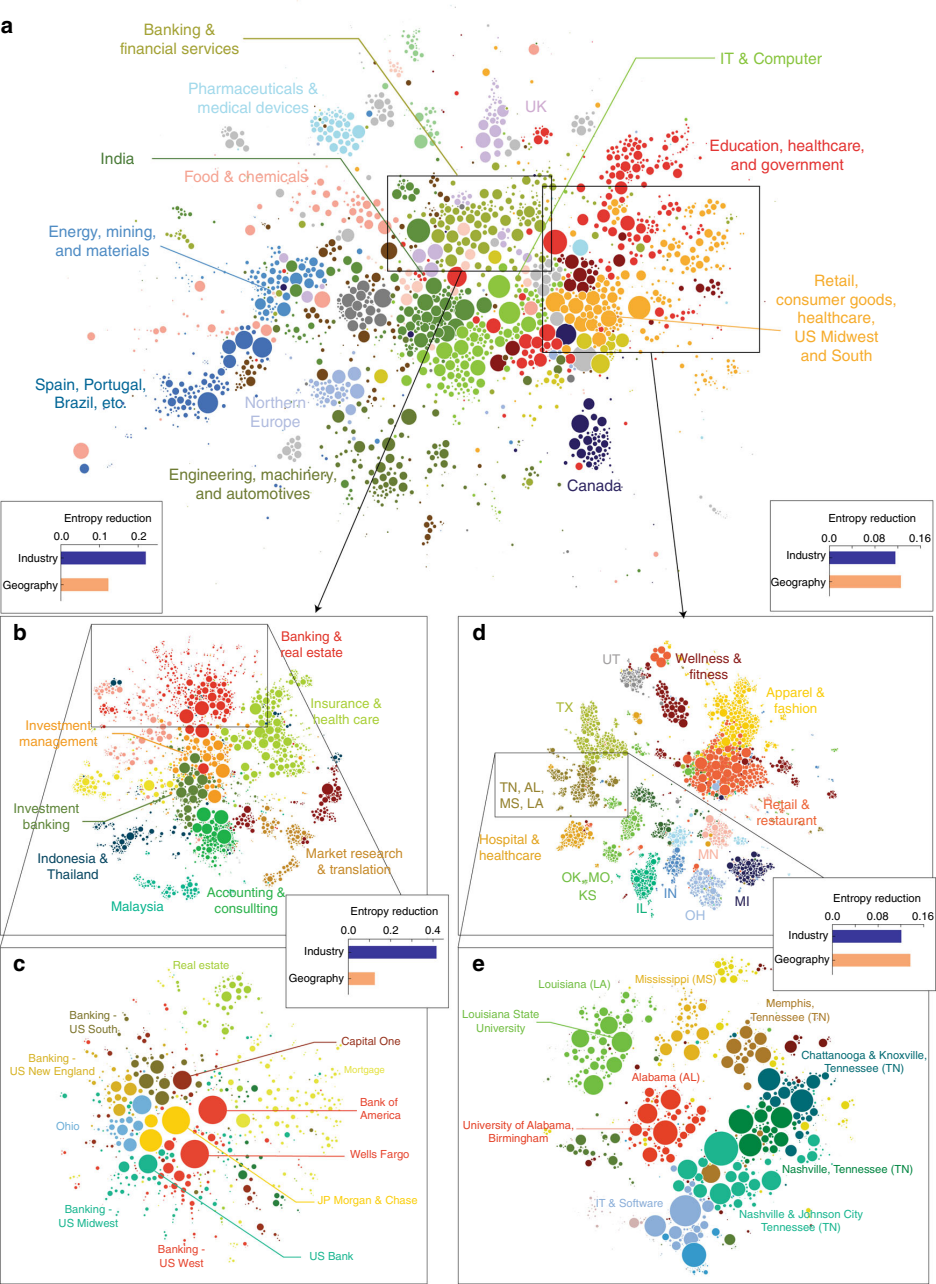
Example of hierarchical structure. **a** The large-scale organization of geo-industrial clusters in the labor flow network. Each circle represents a geo-industrial cluster, with size proportional to its number of employees. The colors represent the highest-level community membership. **b**–**e** Two examples of hierarchical sub-structures in the labor flow network are illustrated. Each circle represents a firm and the bar charts show the reduction in industry and region entropy within the cluster as a proportion of the parent cluster’s entropy. **b**, **c** the organization of banking and financial geo-industrial clusters are affected more by industries than geography. **d**, **e** the geo-industrial clusters in US Midwest and South region form strong geographical clusters

We visualize the network of geo-industrial clusters in Fig. 3a (see Methods for details), where each circle represents a geo-industrial cluster, colored based on the highest-level community membership. We label each highest-level cluster based on the dominant industry or geographical region (See Methods). The map exhibits both industry- and geography-dominated clusters. Cultural and regional economic blocs, such as Northern Europe, stand out, whereas industrial clustering is also evident. For instance, engineering and machinery are associated with automotive clusters, and food production and chemicals are associated with pharmaceutical and medical devices. The map also reveals geographical specializations. Firms located in the Midwest of the United States closely interact with retail and consumer goods industries worldwide, whereas India-based clusters are strongly associated with information technology.

Zooming into lower levels of the geo-industrial hierarchy reveals more intricate structures (See Fig. 3b–e). Two high-level clusters are shown: one focused on banking and financial services in the US, and the other with higher education, healthcare, and retail industries in the US. The banking and financial cluster is broken into more specific industries, such as investment banking and real estate (Fig. 3b). The entropy reduction measure confirms that this hierarchical structure is dominated by industrial categories rather than geographical clustering. On the other hand, the Higher Education, Health Care, and Retail cluster is mostly divided along regional lines. These examples depict the structure of the labor flow network as a complex tapestry of industry and geography.

### Association with economic performance

If geo-industrial clusters can effectively capture both industrial and geographical proximity, can they serve as a useful framework to study the effects of strategic advantage on economic performance? The competition for highly desirable jobs implies that well-educated individuals who are equipped with strong skill sets would be attracted to the sectors and regions that can pay premium wages or rapidly growing ones that may in the future. Furthermore, the industries and regions that attract well-educated people are more likely to benefit from accumulated human capital and spillover effects^33–40^. Motivated by these insights as well as other studies on the effect of labor market integration and knowledge spillover within geo-industrial clusters^12–14^, we examine the labor flow of college-degree workers across regions, industries, and geo-industrial clusters.

**Fig. 4:**
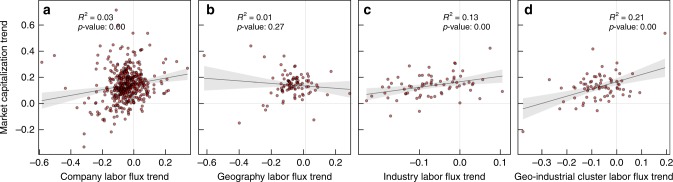
The influx of educated labor force is linked to the growth of geo-industrial clusters. The horizontal axis represents the 5-year trend in college-degree labor flux from 2010 to 2014. Similarly, the vertical axis represents the 5-year trend in log-scaled market capitalization within the cluster over time. **a** The trends for individual firms. **b** The trends for geographical regions. **c** The trends for industries, and **d** the trends for geo-industrial clusters, which displays the strongest relationship

We test how well the influx of educated labor correlates with financial performance when aggregated into different units of analysis. Focusing on the firms in the S&P 500 Index and a time window between 2011 and 2014, we compare their market capitalization growth—measured by the linear temporal trend of log-scaled market capitalization—to the labor flux growth—measured by the linear temporal trend of the log ratio of college-degree labor influx to outflux aggregated in each grouping (see Fig. 4 and Methods).

Overall, we see a positive relationship between the acceleration of college-degree employment growth and market capitalization growth although the strength of the relationship depends on the aggregation used (see Fig. 4). At the level of individual firms, the data is too noisy to establish any clear patterns (Fig. 4a). Geographical aggregation similarly shows little association between labor growth and market capitalization growth, suggesting that location-based grouping is also not a good approach, probably because each location hosts a multitude of disparate industries. Although the industry-level aggregation in Fig. 4c shows a stronger relationship, the strongest correlation can be found in the geo-industrial cluster-based aggregation (see Fig. 4d). These results hold for more complex bayesian models and are robust to the selection of time window, or the inclusion or exclusion of first-job influx and last-job outflux (see Supplementary Figures 3–7, with Supplementary Note 3). The stronger association between the influx of educated labor and economic growth in the geo-industrial cluster level, in comparison with traditional industry- or region-based aggregation, suggests that firms that share labor also share economic growth or decline. This is perhaps driven by shared competitive advantages due to labor market integration and knowledge spillover effects^1,2,12–14^.

### Emerging geo-industrial clusters

**Fig. 5:**
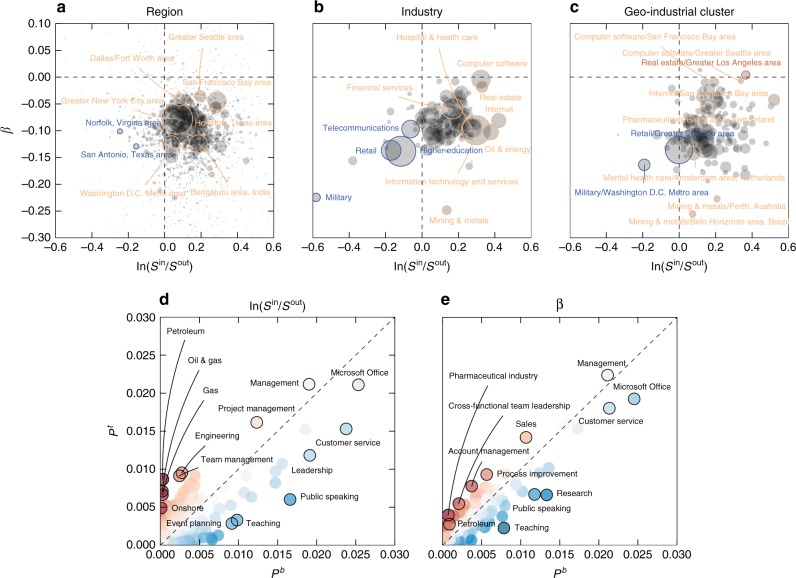
Growth of regions, industries, and geo-industrial clusters and associated skills in growing and declining geo-industrial clusters. **a**–**c** The log-ratio of influx to outflux and its growth over time, aggregated by region **a**, industry **b**, and geo-industrial cluster **c**. The amount of growth is calculated by the log-ratio of influx to outflux (*log*(*S*^in^/*S*^out^)) during each year from 2010 to 2014; its linear time trend (*β*_*i*_) is estimated by the linear regression coefficient of influx ratios to time over this period. The size of a circle represents the number of total transitions either into or out of a corresponding category. **d**, **e** Over-represented skills in geo-industrial clusters in the top and bottom quartiles of log-ratio influx to outflux **d** and its linear time trend **e**. The fraction of people who have a certain skill in the top $\left( {P_q^t} \right)$ and bottom $\left( {P_q^b} \right)$ geo-industrial clusters reveals that specialized and business-oriented skills are more common in growing geo-industrial clusters than declining geo-industrial clusters

We see that the influx of educated workers to a geo-industrial cluster is a meaningful signal of growth, so we can ask which regions, industries, and geo-industrial clusters are seeing that growth. We measure the total growth in terms of influx during a period from 2010 to 2014, using the log ratio of influx to outflux of college-educated workers for each region, industry and geo-industrial cluster, log(*S*^in^/*S*^out^) (See Figure 5a–c and Methods). We then estimate the change of this growth, denoted *β*, by estimating the linear trend in time of the influx log-ratio during the same period. If a region, an industry, or a geo-industrial cluster exhibits a positive net influx and a positive *β*, it means that it has been growing and the growth has been increasing during this period.

Figure 5a shows that most regions are located in the fourth quadrant, with decelerating growth following a strong bounce-back from the Great Recession of 2007–2009^41^. The San Francisco Bay area and the Greater Seattle Area exhibited the strongest growth, whereas places such as San Antonio have been losing educated population. Similarly, most industries also show a slowing growth out of the recession (see Fig. 5b). In this period, the Computer Software industry has been showing the strongest growth, whereas Retail has been losing its educated labor force. This trend has been accelerating. Also note that the Mining & Metals industry has been growing but decelerating, and the Internet and Oil & Energy industries experienced large growth during this period. These employment growth patterns match the relative growth projections from the US Bureau of Labor Statistics’ Occupational Handbook^42^, except that our analysis detects a loss of Retail jobs among the college-educated, and a pronounced deceleration in growth across many fields.

Although these region- and industry-based views paint a rough picture that fits the known recent trends of the global economy, it is the geo-industrial cluster-based analysis that provides the best snapshot of the evolution of the economy. The fact that the San Francisco Bay area has been rapidly growing does not tell us which industry propelled the growth; likewise, the growth of the computer software and internet industries does not inform us where this growth has occurred. By contrast, a cluster-based comparison in Fig. 5c reveals nuanced information about the growth of geo-industrial clusters, completing the picture of economic evolution during this period. The clusters that are based on internet and computer software companies in the San Francisco area, real estate companies in the Los Angeles area, and computer software companies in the Seattle area experienced some of the strongest growth with respect to college-degree workers, while military-related firms and organizations in Washington D.C. and retail companies in the Chicago area experienced the largest decline.

### Emerging skills

This pattern of productivity growth can be supplemented with an even more detailed analysis of associated skills. Here, we identify over- and under-represented skills in emerging and declining geo-industrial clusters (see Supplementary Figure 7 for similar analysis with regions and industries). We compare the aggregated skill distribution of geo-industrial clusters in the top quartile of total influx (log(*S*^in^/*S*^out^); Fig. 5d) or growth (*β*; Fig. 5e) during this period against those in the bottom quartile. The vertical axis represents the fraction of employees with each skill within the top quartile, and the horizontal axis represents the proportion in the bottom quartile. The intensity of the color represents the degree to which each skill is concentrated in the top (red) or bottom (blue) quartile, as measured by the *z* score of the log-odds ratio between the top and bottom skill distributions (see Methods). With respect to the total influx, the over-represented skills in the top geo-industrial clusters are concentrated around management skills, such as Management, Project Management, and Team Management. These results concur with studies on the importance of cognitive-social skills and the prevalence of management-related jobs in high-wage occupations^43–45^. In addition, oil and energy-related skills such as Petroleum, Oil & Gas, Gas, and Onshore are more prevalent in the top quartile, which captures the recent growth of oil and natural gas industry, driven by the new drilling and fracking technologies applied in the US during this period^46–49^.

On the other hand, the most over-represented skills in geo-industrial clusters in the bottom quartile feature widely available, common skills such as Customer service and Microsoft Office, or vague skills such as Leadership. This bias towards common and vague skills in the bottom quartile remains consistent regardless of the focus on the total influx or its growth (Fig. 5e). Although the Leadership skill is more common in the bottom quartile, related, but more specific skills, such as Cross-functional Team Leadership or Process Improvement are over-represented in the top growing geo-industrial clusters. The over-represented skills in the top quartile of influx growth feature newer skills, such as Pharmaceuticals, Biotechnology, and Cloud Computing, capturing new innovations that are attracting educated labor flow.

## Discussion

In this study, we propose a systematic approach to identify geo-industrial clusters by analyzing a massive data set from LinkedIn that captures individual-level labor flow between firms across the world. The map of our geo-industrial clusters is generated organically by high-resolution individual-level data, and allows us to identify the geo-industrial clusters systematically through network community detection, which verifies the importance of region and industry in labor mobility. Also, it shows the relative importance between the two constraints in different hierarchical levels to reveal the practical advantage of the geo-industrial cluster as a unit of future economic analyses.

At the same time, we also note a number of caveats and limitations of our study. Although LinkedIn is widely adopted across the world, the population is still biased towards the US as well as towards a younger population with more technical backgrounds. Moreover, the adoption of LinkedIn is likely to be affected by social diffusion processes, so its data may exhibit stronger clustering and uneven biases. In addition, our approximation uses each firm as a homogeneous unit, which may be inadequate, particularly for large firms that host a wide variety of jobs that are not directly connected to the firms’ main products. Also, we assume geo-industrial clusters are disjoint sets although they are likely to overlap in real world. Finally, our results on the correlation between labor concentration and market capitalization growth are not enough to prove that the influx of educated workers leads to higher valuation, because there may exist other confounding factors, or the direction of causality may be the opposite–higher valuation may lead to more hiring of educated workers. In addition, this analysis focuses only on S&P 500 firms and thus should be interpreted carefully.

We argue that, even with these caveats, the labor flow network approach can provide powerful and novel ways to examine how economies are organized and evolve. Because we focus on the flow between firms, industries, and regions, rather than their size, our results show enough consistency to overcome representation biases. For instance, we expect that the transition matrix in Fig. 1 would be robust against representation biases unless job transition patterns and LinkedIn membership are strongly confounded, and as long as representation bias does not strongly alter the differences between intra- and inter-cluster flow. Finally, as in a previous study on cultural history^50^, focusing on an important sub-population may provide more-meaningful results. Given the high resolution, coverage, and flexibility, we argue that the global labor flow network and geo-industrial cluster framework can serve as a basis for future economic analysis.

Despite its long intellectual history from Alfred Marshall^51^, the hypothesis of the importance of geo-industrial clusters on economic development has only recently become possible to empirically formalize and test through large-scale labor records^16,52^. From this perspective, our study can provide a foundation for further systematic analysis of geo-industrial clusters in the context of business strategy, urban economics, regional economics, and international development as well as providing useful insights for policymakers and business leaders. Furthermore, we expect that future empirical studies on economic geography can compare the labor flow of workers based on their skill levels or gender, in order to contribute to the design of more practical and effective labor policies based on demographic structure.

## Methods

### Labor flow network

A labor flow network is a directed, weighted graph, **G**(**V**, **E**, **W**), in which each node *u* ∈ **V** corresponds to a firm and each edge *e*_*i*→*j*_ ∈ **E** represents the number of individuals who reported employment at firm *i* prior to moving to firm *j* in a given time period (*t*_*s*_, *t*_*e*_). A job transition is included if the start of a job at new firm *j* begins after the start of the time period *t*_*s*_ and before the end of the time period *t*_*e*_, even if the job at *i* was begun before *t*_*s*_. The weight of each edge *w*_*i*→*j*_ ∈ **W** corresponds to the total number of recorded job transitions from firm *i* to firm *j* in the time window. If a member reports multiple job transitions ending or beginning in the same month (the smallest resolution of our time data) a unit weight is divided into all associated transition edges so that $\frac{1}{k}$ is added to each edge, where *k* is the number of edges. The size of firm *i* at time *t*, *s*_*i*_(*t*), is defined by the number of members who reported working at firm *i* at *t*.

We constructed a labor flow network utilizing job history data spanning 1990 to 2015, **G**_(1990,2016)_. We then apply the following procedures to obtain the core of the network: first, removing edges with *w*_*i*→*j*_ < 2; second, 2-core filtering (removing dangling nodes); and third, isolating the largest connected component. This process produces a network representing ~ 42 million job transitions over 8,319,091 edges between 487,782 firms. For yearly analysis, given a year *t* we create a labor flow networks **G**_(*t*,*t* + 1)_ performing no further filtering.

The detailed hierarchical structure is identified by recursively applying the Louvain community detection algorithm^53,54^. We start with the maximum modularity partition (0.47) and keep applying the same method to each community subgraph if the community has >10 nodes. The hierarchical tree that connects each community to its subcommunities is then pruned using metadata, as explained in the following sections.

### Company and cluster feature vectors

Each firm *c* is characterized by a set of firm feature vectors, namely a geography vector **f**^*G*^(*c*) and an industry vector **f**^*I*^(*c*). Each element of these vectors represents the fraction of employees of firm *c* who reported a particular attribute (i.e., a specific region or industry) in their profile. We define the region (industry) of a firm as the most frequent region (industry) in **f**^*G*^ (**f**^*I*^). Similarly, for a given community of firms, *C*, we can describe a cluster feature vector **F**(*C*) where each element represents the fraction of all employees of the firms in the cluster that report that particular attribute.

### Mapping transitions between industry and geographical regions

We construct two transition matrices, one representing labor flows between industries and another representing transitions between the states in the US. In these matrices, each element *T*_*ij*_ represents normalized transition weight from *i* to *j* (*i* and *j* can be either two industries or two regions). The expected flux between *i* and *j* is estimated by

1 $${\Bbb E}(w_{i \to j}) = S_i^{{\mathrm{out}}}\frac{{S_j^{{\mathrm{in}}}}}{{\mathop {\sum}\nolimits_k {S_k^{{\mathrm{in}}}} }},$$

where *S*^out^ is the total number of members who moved out of *i*, and $S_j^{{\mathrm{in}}}$ is the total number of members that moved into *j*. Thus, the normalized flux from *i* to *j* is estimated by

2 $$T_{i \to j} = \frac{{w_{i \to j}}}{{{\Bbb E}(w_{i \to j})}}.$$

As a result, we have *T*_*i*→*j*_ > 1 if there are more people moving from *i* to *j* than expected by the given null model, and *T*_*i*→*j*_ < 1 vice versa.

### Measuring cluster homogeneity

We measure the homogeneity of a cluster using Shannon entropy, a measure of specificity defined for industry vectors by $H^I(C) = - \mathop {\sum}\nolimits_i {F_i^I} (C)\log F_i^I(C),$ where $F_i^I(C)$ represents the element of the cluster feature vector for cluster *C*, corresponding to industry *i*. With geographic entropy, *H*^*G*^ defined similarly using $F_i^G(C)$, the elements of the cluster feature vector for cluster *C*, corresponding to geographical labels *i*.

### Detecting over-represented labels

To identify over-represented industries or geographical regions in a cluster, we employ the log-odds ratio informative Dirichlet prior method^55^. The log-odds ratio of industry or region *w* in cluster *i*, compared with cluster *j* is

3 $$\delta _w^{i - j} = \log \left( {\frac{{f_w^i + f_w^b}}{{N^i + N^b - (f_w^i + f_w^b)}}} \right) - \log \left( {\frac{{f_w^j + f_w^b}}{{N^j + N^b - (f_w^j + f_w^b)}}} \right)$$

where $f_w^i$ is the frequency of *w* in cluster *i*, $f_w^b$ is the pseudo-count for *w* in the Dirichlet prior, *N*^*i*^ is the number of labels in cluster *i*, and *N*^*b*^ is the sum of Dirichlet pseudo-counts. Then the variance and *Z* score are estimated as following:

4 $$\sigma ^2(\delta _w^{i - j}) \approx \frac{1}{{f_w^i + f_w^b}} + \frac{1}{{f_w^j + f_w^b}},Z = \frac{{\delta _w^{i - j}}}{{\sqrt {\sigma ^2\left( {\delta _w^{i - j}} \right)} }}$$

We make an approximation by considering all other clusters as the other cluster (*j*) and the set of all firms as the background corpus.

### Metadata-based pruning

We employ a metadata-based stopping heuristic for recursive community detection to identify a particular partition from the hierarchical structure. Our main idea is that we can safely split a community if it can be broken into multiple communities, each of which exhibits strongly over-represented industry or geographical region metadata, and that such splitting is inappropriate if the resulting children do not have any over-represented metadata. Our method moves down the tree from the root to the finest level of the community hierarchy that maintains significant over-representation of particular regions or industries within the community. Given two thresholds, *θ*_*b*_ (break threshold) and *θ*_*k*_ (keep threshold), we look at whether the current community over-represents some industry and region label, with *Z* score surpassing *θ*_*b*_. If it does not or is a leaf-node, we keep the community if it over-represents some industry and region label with a more lenient keep threshold *θ*_*k*_, and otherwise prune the community from the tree. If it does over-represent metadata at or above the threshold of *θ*_*b*_, the process is repeated for the community’s children. This algorithm is laid out in Algorithm 1. We use *θ*_*k*_ = 1.96 and *θ*_*b*_ = 100 for financial data analysis, as this threshold provided a moderate number of communities, without pruning any firms for which we had financial data. This pruned set of communities (looking only at the subgraph of companies within them) have a modularity of 0.407. We use *θ*_*b*_ = 10 for visualizations.

Algorithm 1 Pruning to minimum threshold **Require**: *tree*, *root*, *θ*_*k*_, *θ*_*b*_

**Ensure**: *save*_*list*

1. *visit*_*list* ← *root*
2. *save*_*list* ← *list*()
3. **for** *node* ∈ *visit*_*list* **do**
4.  *children* ← *tree*.*children*(*node*)
5.  **for** *child* ∈ *children* **do**
6.   **if** *child*.*max*_*Z*_*score* > *θ*_*k*_ for industry and region **then**
7.    *visit*_*list*.*append*(*child*)
8.   **else if** *child*.*max*_*Z*_*score* > *θ*_*b*_ for industry and region **then**
9.    *save*_*list*.*append*(*child*)
10.   **end if**
11.  **end for**
12. **end if**
13. **return** *save*_*list*

### Entropy reduction

We measure the entropy of industry and geographical region cluster feature vectors at each level of the cluster hierarchy to validate our community detection strategy as well as to compare the impact of geography and industry in job transitions. Entropy reduction as shown in Fig. 2 is calculated for both industry and regional labels as a ratio of the difference between the global entropy *H*(*V*) and a community *j*'s entropy *H*(*j*) to the global entropy, $d_j = \frac{{H(V) - H(j)}}{{H(V)}}$. This ratio is used instead of the raw entropy reduction to provide a comparable scale between industry and geographical region metadata, as there are many more possible region labels than industry labels. $\rho _k = |\{ j|d_j^I{\,} > {\,} d_j^G;j \in K_k\} |/|K_k|$ is the proportion of communities *j* in the set of communities *K*_*k*_ at level *k* of the hierarchy with a greater reduction in industry entropy *d*^*I*^ than geographical entropy *d*^*G*^. The average entropy reduction over all communities in each hierarchical level weighted by the number of firms is reported as $\bar d_k = \frac{{\mathop {\sum}\nolimits_{j \in K_k} w_j \cdot d_j}}{{\mathop {\sum}\nolimits_{j \in K_k} w_j}}$ where *w*_*j*_ is the number of firms in community *j*—and its standard error is estimated by Cochran’s method as reported in a previous study^56^. This is equivalent to the mutual information between community and industry or geography partitions at each level of the hierarchy, normalized by the overall industry or geographical entropy. This is an imperfect measure (and there may be no perfect measure for clustering comparisons^57^), which still favors comparisons between sets with more possible labels^58^, such that we are likely over-estimating the importance of geography, but it does allow for some comparison. We employ a tree-shuffling null model that randomly shuffles all firms throughout the hierarchical community tree such that the tree is still a consistent community hierarchy; for each firm *c*, a firm $\hat c$ is randomly selected from the set of all firms, and *c* is replaced by $\hat c$ firm in each community to which it belonged, giving us corresponding null values $\bar d_k^\prime$ with the difference $\Delta _k = \bar d_k - \bar d_k^\prime$.

### Marketcap trends

We use the market capitalization data for S&P 500 firms from 1996 through 2015. For each given partition (i.e., geographical regions, industries, and selected geo-industrial clusters), we aggregate all market capitalization within a cluster by summing them. The influx and outflux are also aggregated at the cluster level, ignoring within-cluster flow, but including first recorded jobs as influx and last recorded jobs as outflux. To find trends over time, we performed a ordinary least-squares linear regression between a variable representing time and the variable of interest as shown below:

5 $${\mathrm{MC}}_{i,t} = \beta _{{\mathrm{MC}},i}t + \mu _{{\mathrm{MC}},i}$$

6 $${\mathrm{LF}}_{i,t} = \beta _{{\mathrm{LF}},i}t + \mu _{{\mathrm{LF}},i}$$

where MC_*i*,*t*_ and LF_*i*,*t*_ are the quarter-four log market capitalization and yearly labor flow respectively for cluster *i* at time *t*, *β* is the slope of the regression, and *μ* is the intercept. The slope of the regression is then used as the trend *β* in the further regression:

7 $$\beta _{{\mathrm{MC}},i} = \beta _\beta \beta _{{\mathrm{LF}},i} + \mu _\beta$$

Although this model is intuitive, it treats inferred parameters as observed. A more complete Bayesian model that also accounts for errors in parameter estimation is included in Bayesian Model for Trends of Trends in Supplementary Note 3.

## Supplementary information

Supplementary Information

## Data Availability

The data that support the findings of this study are available from LinkedIn but restrictions apply to the availability of these data, which were used under license for the current study, and so are not publicly available. However, replication data are available from LinkedIn upon reasonable request and with permission. Researchers wishing to reproduce research should submit a request to egr_data@linkedin.com.

## Code availability

The Python code for our recursive Louvain community detection algorithm and metadata-based pruning are available on our GitHub repository (https://github.com/jaehyukpark/global_labor_flow_network).
