# Supplementary material for: Global labor flow network reveals the hierarchical organization and dynamics of geo-industrial clusters
Source: Nat Commun. 2019 Aug 1;10:3449. doi: 10.1038/s41467-019-11380-w (PMC6671949; doi:10.1038/s41467-019-11380-w)
Supplement: Supplementary file 1 — Supplementary Information [file 41467_2019_11380_MOESM1_ESM.pdf]

Supplementary Information: Global labor flow network reveals the hierarchical organization and dynamics of geoindustrial clusters

Park *et al.*

Supplementary Figures

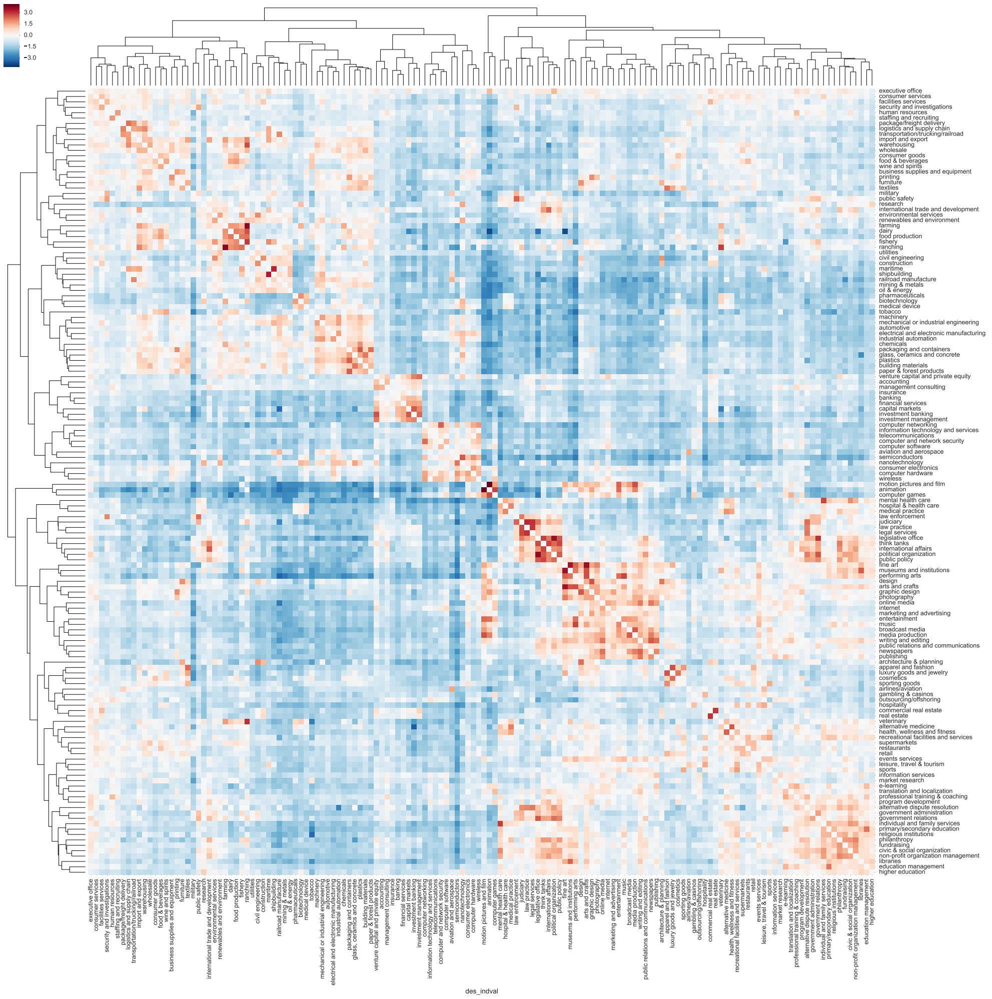

Supplementary Figure 1: Labor flow between LinkedIn industries. Red represents transitions where the flow between pairs of industries are more than expected, and blue colors represent less-than-expected. Hierarchical clustering is used to generate the clusters.

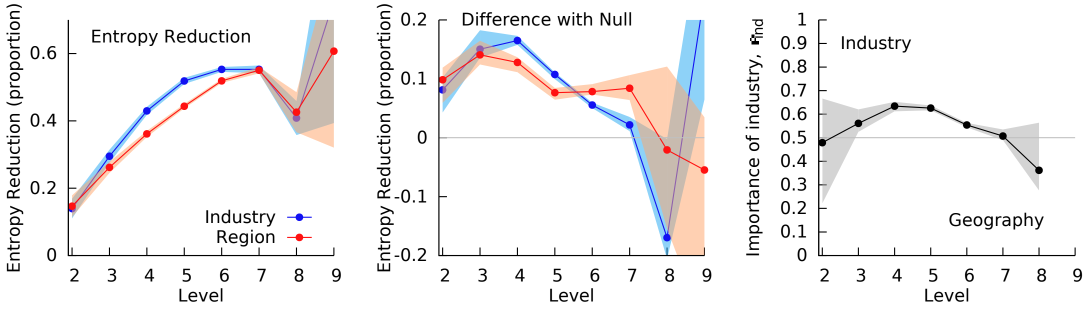

Supplementary Figure 2: Entropy reduction across all levels. This figure shows the entropy reduction across all levels of the hierarchy, corresponding to Figure 2 in the main paper, but including the noisy last two levels of the hierarchy

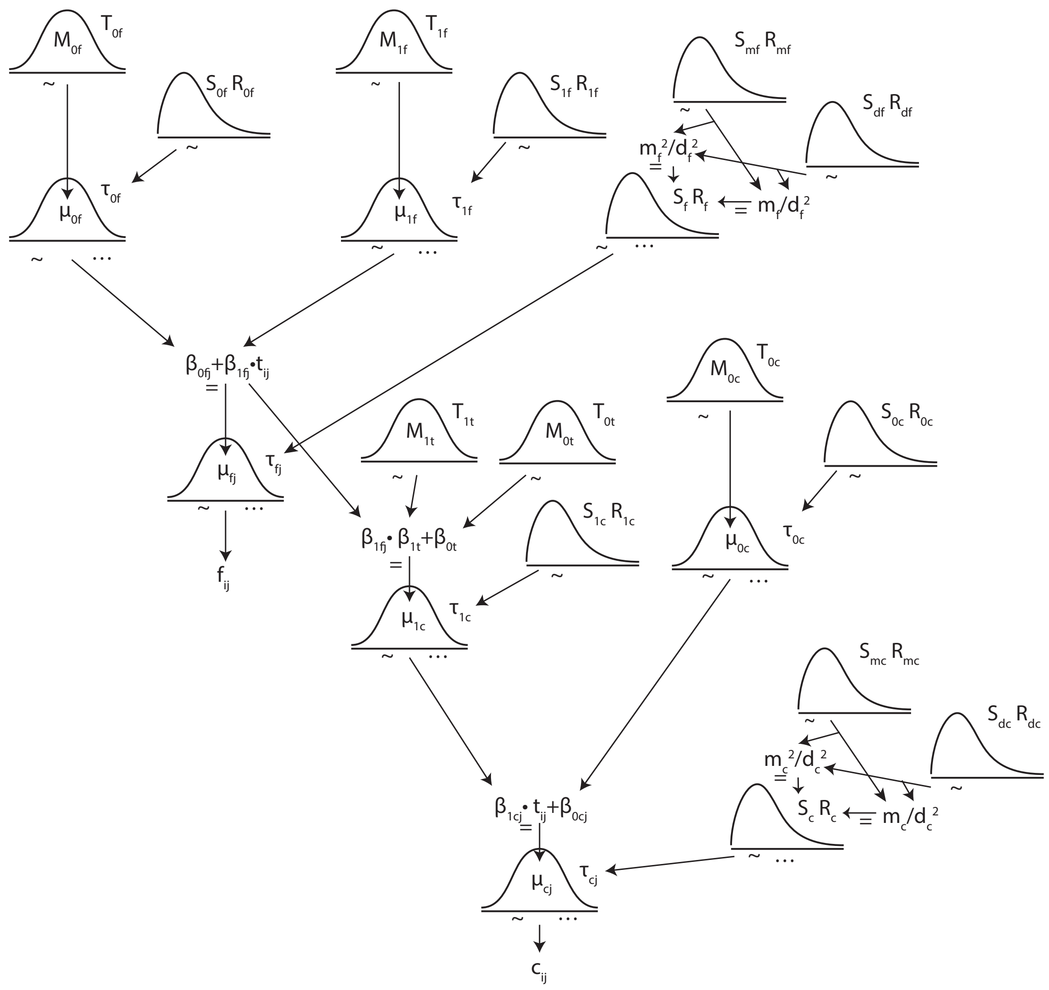

Supplementary Figure 3: Model Diagram. Hyperprior constants were all set to be somewhat uninformative: $M = 0$, $T = 0.01$, $S = 1$ and $R = .1$. The data is standardized to normal z-scores before the MCMC sampling. The initial point to start the MCMC chain is set by taking the mean and standard deviations of the data, and performing the linear regressions separately to initialize β. 500 steps are allowed for burn-in, and 50,000 steps are saved as posterior samples.

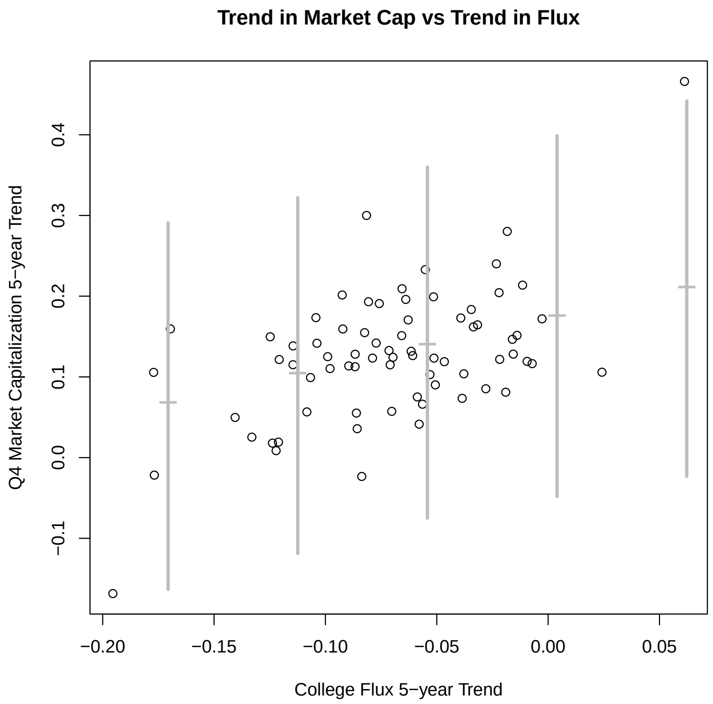

Supplementary Figure 4: Trend of Trends Regression. This figure shows the middle regression $\beta_{1,c,j} = \beta_{1,f,j} * \beta_{1,t} + \beta_{0,t}$ for our found clusters during the time period between 2010 and 2014. The y-axis is the mean posterior estimate of $\beta_{1,c,j}$, and the x-axis is the mean posterior estimate of $\beta_{1,f,j}$ used in the time regressions. Gray bars represent the 95% confidence intervals around the mean estimate for the linear regression.

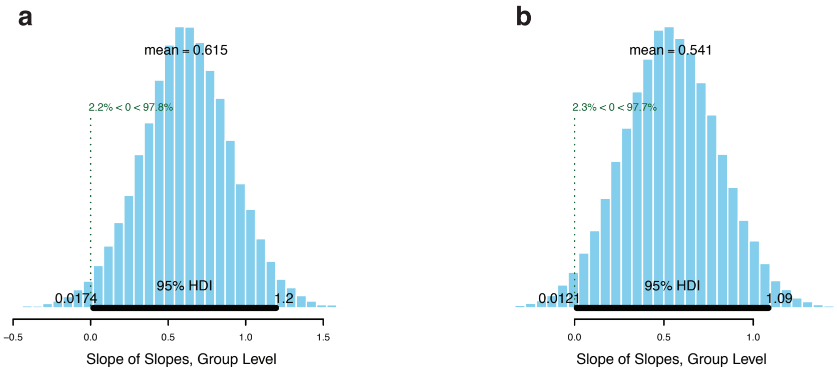

Supplementary Figure 5: Posterior slope $\beta_{1,t}$. **a**, estimation for our clusters, **b**, estimation for industry lables.

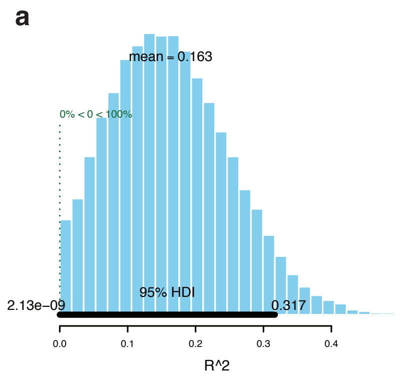

Supplementary Figure 6: Posterior R squared estimates. **a**, estimation for our clusters, **b**, estimation for industry labels

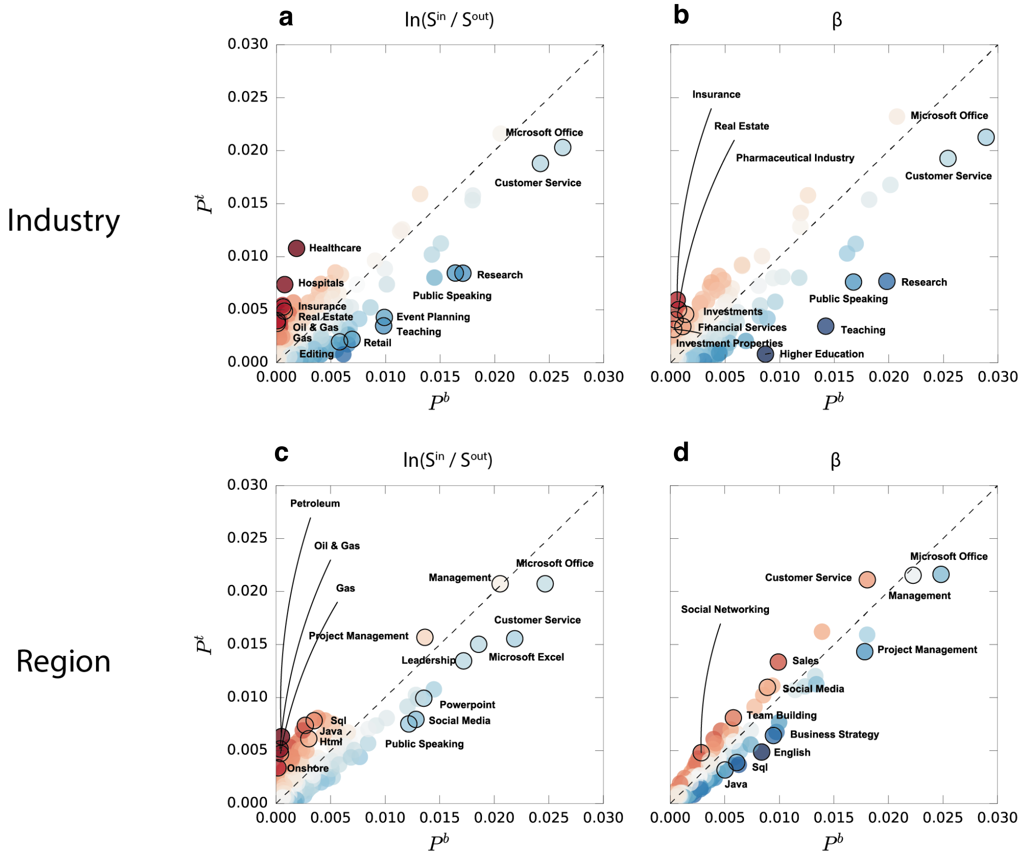

Supplementary Figure 7: Overrepresented skills in the top and bottom quartile industries (**a** and **b**) and regions (**c** and **d**). The fraction of people who have a certain skill in the top (P_q^t) and bottom (P_q^b) geo-industrial clusters shows which skills are more common in growing geo-industrial clusters compared to declining geo-industrial clusters. Top and bottom quartiles are decided based on the total flux ratio ($\ln(S^{in}/S^{out})$) during the period (**a** and **c**) or the regression coefficient of the flux ratio growth (β_i) over the time period (**b** and **d**).

Supplementary Notes

Supplementary Note 1. Labor Flow between Industries

To reveal the macro-scale structure of the labor flow network, we examine the flow between industries by aggregating companies based on their industries. Although companies list LinkedIn industry codes in their metadata, we do not use this industry code, as it is entered by the person who manages the LinkedIn profile of the company, and can be inaccurate depending on the profile manager. Instead, we use a more organic method to determine the industry of each company — using the industry information of the employees. Not only the companies, but also the members are asked to document their industry by choosing a code from LinkedIn's industry classification. For each company, we aggregate all employees' industry codes and consider the most frequent industry code as the industry code of the company. Of the 4,152,815 companies in our labor flow network, 2,542,817 (61%) have a majority of employees on LinkedIn who agree with the company page's industry code.

Using the industry code defined as explained above, we create a transition matrix that describes the labor flow between industries. Supplementary Figure 1 shows the flux among LinkedIn's industry categories. In this figure, the rows are the sources of the transition, columns are the destinations, and the colors of cells represent the weight of links between each pair of nodes, normalized with the method described in Methods. Clusters are produced with hierarchical clustering, with linkage method = 'single', threshold = 1.15. The time window of data used is 1990-2015.

Supplementary Note 2. Full Entropy Reduction

Supplementary Figure 2 shows the entropy reduction plots from Figure 2 of the main text for all levels of the hierarchy. The bottom two levels were removed from the main text because they are clear over-partitions of the data. Level 8 is too noisy to draw any detailed conclusions because it is composed of 1,477 companies split into 495 communities. Level 9 is a partitioning of only one of the Level 8 communities, containing only 11 companies split into eight communities.

Supplementary Note 3. Bayesian Model for Trends of Trends

Here we describe a Bayesian model to capture a correlation between two trends. This model is diagrammed in Supplementary Figure 3. There are two variables of interest: f, the "flux" of college educated individuals moving into or out of a cluster, defined as the log ratio of the influx to outflux for that cluster; and c, the summed log market capitalization of S & P 500 companies in that cluster. In addition, there is a time variable t, the year of the corresponding data, and two data indexes: i, a time index, and j, a cluster index. The general idea of the model is to find the linear time trends of f and c, and then find the linear relationship between the two trends. This was initially performed using three separate regressions. First:

$$c_{i,j} = \beta_{1,c,j} \times t_{i,j} + \beta_{0,c,j} \tag{1}$$

$$f_{i,j} = \beta_{1,f,j} \times t_{i,j} + \beta_{0,f,j} \tag{2}$$

Here, the $\beta_{1,c,j}$ and $\beta_{1,f,j}$ represent the linear trend in time for capitalization c and flux f for cluster j. The relationship between trends is captured in a further regression:

$$\beta_{1,c,j} = \beta_{1,f,j} \times \beta_{1,t} + \beta_{0,t} \tag{3}$$

However, if we conduct these regressions separately, we are essentially inferring one parameter twice, $\beta_{1,c,j}$, once as a coefficient and once as the dependent variable in a linear regression, and ignoring differences in error. As an alternative, we can create the model shown in Supplementary Figure 3. Here, all three regressions are performed, but the $\beta_{1,f,j}$ coefficient is explicitly used as both a coefficient in the time trend regression on f and to generate the distribution (group level prior) from which $\beta_{1,c,j}$ is drawn. Group level priors are added to all coefficients, and individual clusters are given their own error estimations.

Supplementary Figure 5 show the posterior distribution of the slope $\beta_{1,t}$ and 6 shows the posterior distribution of R-squared values for our found clusters and clusters defined by industry labels. Both slopes are confidently non-zero.
